# Delayed Intervention of Small Renal Masses on Active Surveillance

**DOI:** 10.15586/jkcvhl.2017.75

**Published:** 2017-05-24

**Authors:** Mohit Gupta, Michael L. Blute, Li-Ming Su, Paul L. Crispen

**Affiliations:** Department of Urology, University of Florida College of Medicine, Gainesville, FL, USA

**Keywords:** active surveillance, delayed intervention, renal cell carcinoma, renal mass, small renal mass

## Abstract

Although surgical excision is the standard of therapy for small renal masses (SRMs), there is a growing recognition of active surveillance as an option in select patients who are poor surgical candidates or who have shorter life expectancy. A number of patients on expectant management, however, subsequently advance to definitive therapy. In this study, we systematically reviewed the literature and performed a pooled analysis of active surveillance series to evaluate the rate and indications for definitive treatment after initiating a period of active surveillance. Fourteen clinical series (1245 patients; 1364 lesions) met our selection criteria. Mean lesion size at presentation was 2.30 ± 0.40 cm with a mean follow-up of 33.6 ± 16.9 months. Collectively, 34.0% of patients underwent delayed intervention, which ranged in individual series from 3.6% to 70.3%. Of patients undergoing delayed intervention, the average time on active surveillance prior to definitive treatment was 27.8 ± 10.6 months. A pooled analysis revealed that 41.0% of patients underwent therapy secondary to tumor growth rate and 51.9% secondary to patient or physician preference in the absence of clinical progression. Overall, 1.1% of all patients progressed to metastatic disease during the average follow-up period. Thus, active surveillance may be an appropriate option for carefully selected patients with SRMs. However, delayed treatment is pursued in a significant percentage of patients within 3 years. Prospective registries and clinical trials with standardized indications for delayed intervention are needed to establish true rates of disease progressions and recommendations for delayed intervention.

## Introduction

The presentation of a patient with a small, incidentally found renal lesion (<4 cm) is now a common clinical encounter for practicing urologists. Due to the increased use of diagnostic cross-sectional imaging, small renal masses (SRMs) are being identified with greater frequency and now account for 48%–66% of renal cell carcinoma (RCC) diagnoses (1). This has led to an increased incidence of RCC during the last three decades and a concurrent increase in the rates of surgical intervention (2, 3). Despite earlier diagnosis and treatment, however, there has not been a significant increase in cancer-specific survival (CSS) or overall survival (OS) for patients with SRMs (2–5).

For clinically localized SRMs, current treatment options include surgical excision, ablation, and/or active surveillance (AS) based on emerging evidence demonstrating equivalent, short- and intermediate-term cancer-specific outcomes among these modalities (2). Survival following surgical excision is well established with 5-year CSS rates over 95% following partial or radical nephrectomy for patients with pT1a lesions (4, 6). Although a number of ablative technologies have emerged as potential treatment options, data on the long-term effectiveness of these ablative techniques as valid alternatives to surgery have yet to be clearly demonstrated (2). Recent studies have suggested CSS survival rates approaching those achieved with surgical excision; however, the majority of the series evaluating these techniques have enrolled too few patients and employed disparate follow-up protocols to yield meaningful conclusions (7).

While surgical therapy remains the cornerstone of treatment for SRMs, the role of AS has also gained acceptance as an option for select patients. These patients may be poor surgical candidates due to age or competing health risks, or they may be unwilling to accept the risks associated with surgical management (3, 8). Poor performance status may limit their ability to handle the physiologic stress of surgery, and preexisting medical comorbidities such as cardiovascular disease and chronic kidney disease may attenuate the survival benefit rendered by surgical intervention. In addition, recent studies have shown that patients over 75 years of age are more likely to die of cardiovascular and other non-cancerous comorbidities than their clinically localized renal mass (3, 8, 9).

Use of AS has become more palatable in part from a greater understanding of the biology of localized renal tumors. While the natural history of SRMs is heterogeneous, multiple studies have found that the majority of tumors under AS demonstrate a slow interval growth and have a low rate of metastatic progression (5, 10, 11). A meta-analysis of nine recent AS series revealed a mean growth rate of 0.28 cm/year in 234 observed tumors over a median follow-up of 32 months. While growth rates of confirmed RCC lesions varied considerably (rate 0.42 to 1.6 cm yearly), progression to metastatic disease was identified in only 1% of lesions (12).

Of the patients who initiate AS, a notable subset will eventually undergo definitive treatment. Retrospective studies have varied substantially in citing rates of patients who progress to delayed intervention (3.6%–70.2%) (13, 14). Data regarding the reasons, timing, and indications for subsequent definitive treatment after initiating a period of AS are heterogeneous and remain limited (12). In this article, we consequently reviewed contemporary studies on AS of SRMs and pooled data from individual series to evaluate the rate of and indications for progression to delayed surgical intervention.

## Materials and methods

We performed a literature search of English-language publications in the MEDLINE database to identify clinical studies that reported AS of clinically localized SRMs from 1995 to 2016 using the National Center for Biotechnology Information’s PubMed site. Studies on SRMs that were clinically localized at initial presentation were included. Series that included metastatic RCC and those that did not differentiate the growth rates of localized versus metastatic disease were excluded from our study. Studies analyzing duplicate or previously studied populations were excluded. Furthermore, case reports on the observation or treatment of single lesions were excluded from analysis.

Pathological data from individual series were pooled to evaluate the rate of and indications for delayed intervention. Variables extracted included number of lesions, mean age of presentation, mean tumor size (in cm), mean growth rate per year (in cm per year), mean duration of AS, percentage of patients who underwent delayed intervention, percentage of solid versus cystic masses, and percent of metastatic progression. Overall weighted mean estimates were calculated for these variables by combining data from individual series with complete information. A further subset analysis was performed to evaluate characteristics of lesions that were treated with delayed intervention versus those that were kept on AS protocol.

## Results

**Figure 1:**
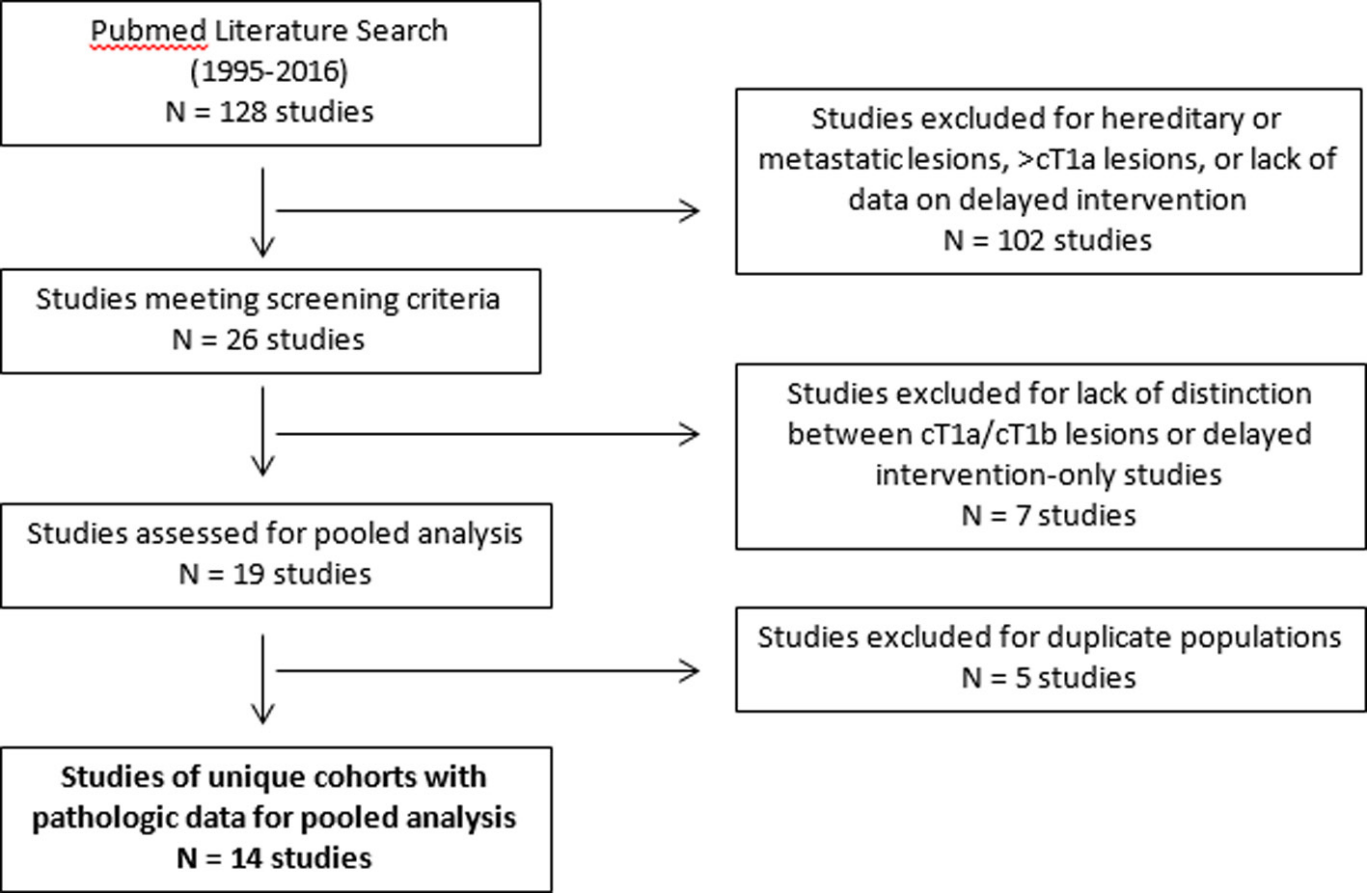
Study selection flow diagram.

**Figure 2:**
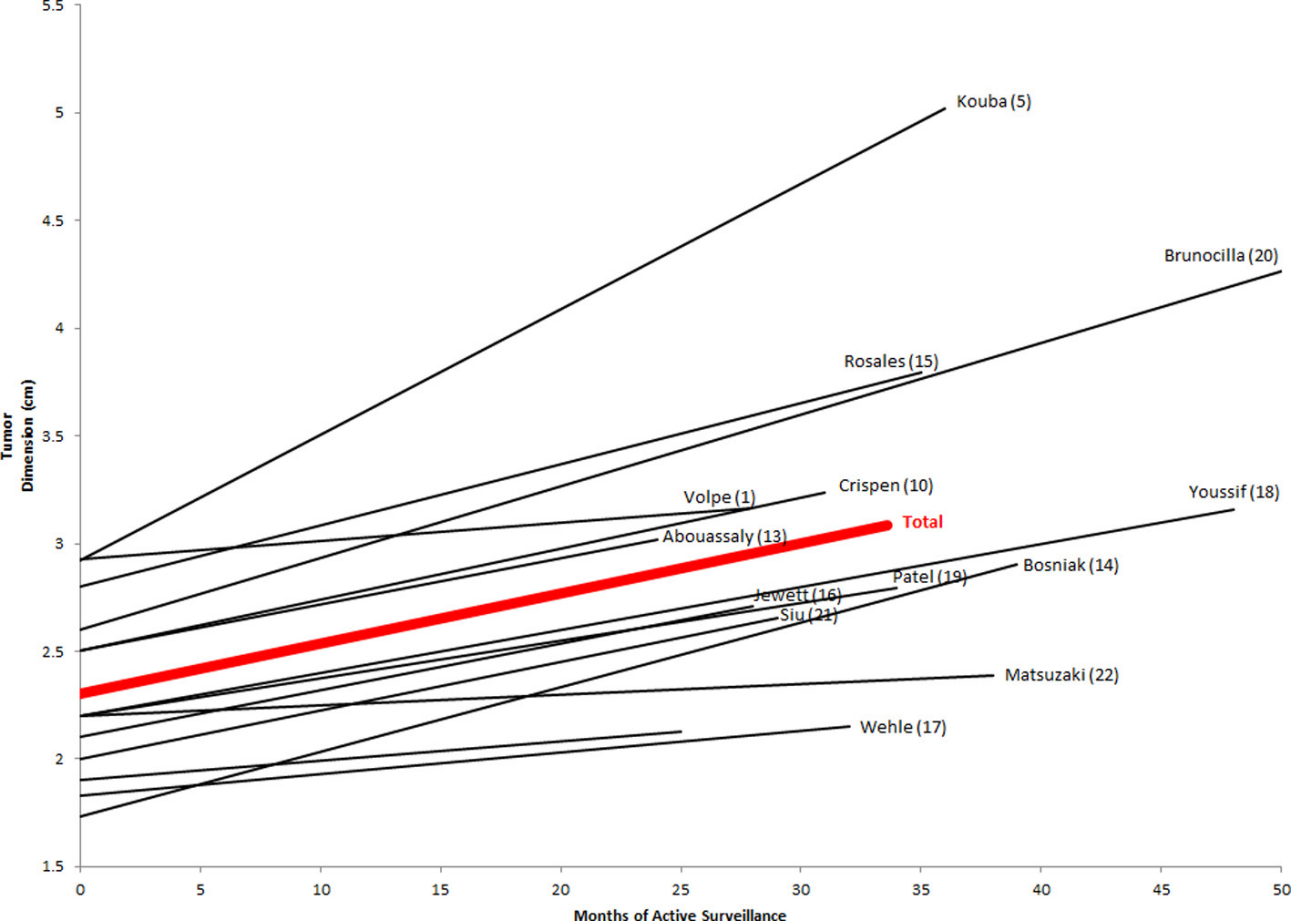
Tumor dimension versus time on active surveillance. Line slopes represent mean growth rate in each series.

Our systematic literature review yielded a total of 14 clinical series of unique cohorts (Figure 1) that met our inclusion criteria (1, 5, 10–22). Collectively, these studies accounted for a total of 1245 lesions and 1364 patients. The mean number of lesions followed in each study was 86 (median 55, range 15–240). A pooled analysis (Table 1) of cohort studies revealed a mean age (± standard deviation) of 71.9 ± 3.9 years. Mean tumor dimension at presentation was 2.30 ± 0.40 cm, and among tumors for which pathologic data were available, 92.6% of lesions were characterized as solid and the remainder were classified as Bosniak IV cysts. Overall, there was a mean follow-up of 33.6 ± 16.9 months for all patients. Lesions had a mean growth rate of 0.26 ± 0.16 cm per year (Figure 2). Diagnostic biopsy was utilized in 21.8% of masses. Four of 14 studies provided quantifiable indications for selecting AS. In sum, 34.0% of patients subsequently underwent delayed intervention, and rates of delayed intervention in individual series ranged from 3.6% to 70.3%. Overall, 1.1% of all patients progressed to metastatic disease during the mean follow-up period. Of the series with available outcome data, 11.9% of patients died of any cause and 1.2% died of metastatic RCC.

**Table 1:** Retrospective studies of unique cohorts of small renal masses managed on active surveillance with available pathologic data.

Study	Year	Number of patients/SRMs	Mean age (years)	Mean initial tumor dimension (cm)	Mean linear growth rate (cm/year)	Mean surveillance follow-up (months)	Metastatic progression (%)	Delayed intervention (%)
Bosniak (14)	1995	37/40	66	1.73	0.36	39	0.0	70.3
Volpe (1)	2004	29/32	71	2.93	0.10	28	0.0	27.6
Wehle (17)	2004	29/29	70	1.83	0.12	32	0.0	31.0
Siu (21)	2006	47/47	68	2.00	0.27	29	2.0	29.8
Kouba (5)	2007	43/46	67	2.92	0.70	36	0.0	30.2
Matsuzaki (22)	2007	15/15	67	2.20	0.06	38	0.0	20.0
Youssif (18)	2007	35/44	72	2.20	0.24	48	5.7	22.9
Abouassaly (13)	2008	110/110	81	**2.50**	**0.26**	**24**	0.0	3.6
Crispen (10)	2009	154/172	69	2.50	0.29	31	1.3	44.2
Rosales (15)	2010	212/223	71	**2.80**	**0.34**	**35**	1.9	5.2
Jewett (16)	2011	178/209	73	2.10	0.26	28	1.1	12.9
Patel (19)	2011	71/93	72	2.20	0.21	34	1.4	19.7
Brunocilla (20)	2013	62/64	75	**2.60**	**0.70**	**92**	3.2	25.8
Pierorazio (25)	2015	223/240	71	**1.90**	**0.11**	**25**	0.0	9.4
Totals		1245/1364	71	2.30 ± 0.40	0.26 ± 0.16	33.6 ± 16.9	1.1	34.0

Bold font signifies median values provided in the study.

**Figure 3:**
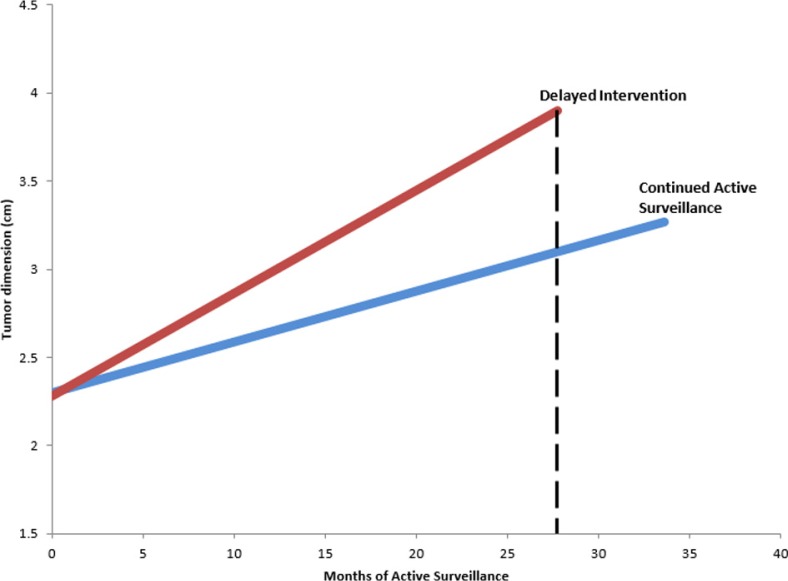
Tumor dimension versus time on active surveillance for patients undergoing delayed intervention versus those on active surveillance. Line slopes represent mean growth rate in each series. Dashed line represents mean surveillance time.

Of patients undergoing delayed intervention, a subset analysis of available data demonstrated the average time on AS prior to definitive treatment was 27.75 ± 10.57 months. Patients undergoing delayed intervention were generally younger compared with other patients in AS (64.2 vs. 71.9 years old, respectively). The mean growth rate of tumors that eventually underwent intervention was 0.70 ± 0.61 cm per year (Figure 3). In comparison, tumors that remained on AS demonstrated a mean growth rate of 0.28 ± 0.20 cm per year. Twelve of 14 studies provided indications for delayed therapy. Our pooled analysis revealed that 41.0% of patients underwent therapy secondary to tumor growth rate and 51.9% secondary to patient or physician preference in the absence of clinical progression. The remainder of patients underwent therapy secondary to symptomatic progression. Of the series with available subset data, 29.9% of patients who underwent delayed surgical intervention underwent radical nephrectomy and 70.1% underwent a nephron-sparing approach such as partial nephrectomy or thermal ablation. Based on this subset, 8.3% of patients who underwent delayed treatment died of any cause during the follow-up period.

## Discussion

Patients with clinical stage I renal masses have traditionally undergone surgical removal with curative intent. The primary goal of treating these often-incidental lesions has been to provide oncologic control and alter the natural course of the malignancy (10). Treatment of these lesions has not, however, yielded a decrease in cancer-specific deaths from RCC, suggesting the possible overtreatment of small, potentially indolent renal tumors (3). Furthermore, there is growing recognition that competing risks from comorbidities in elderly or infirm patients may outweigh the survival benefits otherwise rendered by surgical intervention in patients with localized renal tumors (8). For these reasons, AS of small renal tumors is now a recognized option for patients who have limited life expectancy (23).

Retrospective cohort studies have helped provide an understanding of the natural history of SRMs. Chawla et al. (12) noted the risk of progression of SRMs to metastatic disease while on AS was low (1%). Another meta-analysis by Kunkle et al. (2) of 99 series and 6471 renal lesions managed with surgery, ablation, or AS noted no statistical differences in the incidence of metastatic progression regardless of how tumors were managed. These results suggest that AS is a safe treatment alternative for patients that are elderly or have significant, competing medical comorbidities.

Current data, however, suggest that delayed treatment is pursued in a significant percentage of patients within 3 years. Thresholds for termination of an AS protocol and implementation of definitive intervention, however, are unclear and not well defined in the majority of published series. Typically, the decision to proceed with delayed intervention is based largely upon tumor growth rate or changes in the radiographic characteristics of the tumor. In our pooled analysis, an increase in tumor size was cited as the primary reason for 41.01% of patients who underwent delayed intervention. As our study reveals, mean observed tumor growth rate was notably greater in patients who eventually underwent delayed intervention compared with patients who remained on surveillance (0.70 cm/year vs. 0.28 cm/year). This finding suggests that a tumor’s observed growth kinetics can weigh heavily upon physicians’ and patients’ decision to pursue delayed intervention. It is unclear from current literature whether tumor growth kinetics, however, are associated with unfavorable biological activity, and a direct correlation is yet to be demonstrated (12). Kunkle et al. (24), for example, found that a substantial proportion (26%–33%) of renal tumors exhibited zero radiographic growth over a median 29-month follow-up and reported similar rates of malignancy compared with growing lesions. Recent studies have also found that the rate of tumor growth has not been shown to predict malignant histology in tumors which do demonstrate interval growth (12, 21). Furthermore, a multicenter prospective study by Jewett et al. (16) *also* showed that histologically proven benign tumors (e.g., angiomyolipoma and oncocytoma) appear to grow at the same rate as malignant ones. Our study was unable to corroborate these findings due to limitations of histologic data in our clinical series.

Our pooled analysis also revealed 51.9% of patients who elected delayed intervention did so secondary to patient or physician preference in the absence of clinical progression. This was based largely upon patient or physician perceptions of risk of remaining on AS or upon a change in a patient’s medical clearance by his or her physician. In the absence of curative treatment options for metastatic disease, patients and physicians who elect AS assume a calculated risk of the development of metastasis during or after the AS period (18). Patient anxiety about disease progression and of potential limitations on available treatment options in the future can consequently influence their decision to terminate AS. Recent series, however, have suggested that a period of AS followed by delayed primary intervention is not associated with alterations in treatment plan or stage migration (5, 25). Some authors have also shown that delaying intervention on AS does not limit or compromise the feasibility of nephron-sparing surgery nor does it lead to an increased risk of local or metastatic progression (25, 26).

Limitations of our study stem from the predominantly retrospective nature of the studies included in our analysis. The majority of the studies had a short duration of follow-up (mean 33.6 months) and provided little data on how AS impacted patient quality of life. In addition, there was a lack of uniform indications for initiating or terminating AS among the studies included. One report included in our analysis was prospective in nature: Pierorazio et al. (27) recently published the results from the multi-institutional delayed intervention and surveillance for SRMS (DISSRM) registry for 497 patients with SRMs who prospectively underwent AS versus primary intervention (PI). Over a median follow-up of 2.1 years, 9% of patients on AS underwent delayed intervention. They found that AS was not inferior to PI for a well-selected cohort of patients: OS for PI and AS was 98% and 96% at 2 years, and 92% and 75% at 5 years, respectively (log rank, *P* = 0.06). At 5 years, CSS was 99% and 100% for PI and AS, respectively (*P* = 0.3). Of the patients that underwent delayed intervention, 71% were elective or patient preference (growth rate 0.08 cm/year) and 29% were due to growth rate >0.5 cm/year (mean growth rate 1.1 cm/year). These results support the notion that patient concern for disease progression plays a significant role in the duration of AS and decision to pursue delayed intervention. While this study suggests that tumor growth rate during AS is a common indication for terminating AS, data from the DISSRM registry suggest that the number of patients terminating AS due to tumor growth rate may be decreased by establishing thresholds for intervention. Data from the DISSRM registry also demonstrated that there was no difference in quality of life assessments between patients undergoing AS versus primary intervention; AS of SRMs did not appear to negatively impact patients’ quality of life or mental health (28).

Our study suggests that delayed surgical treatment is pursued in a significant percentage of patients within 28 months of initiating AS. While the risk to metastatic progression is low, AS may not be a durable option for patients who are acceptable surgical candidates or who have extended life expectancies. AS should consequently only be used as alternative to definitive therapy in select patients who have limited life expectancy or competing health risks that preclude surgery. Appropriate patient counseling prior to initiating AS is therefore paramount in establishing whether AS is a suitable treatment modality for managing a patient’s SRM. If AS for SRMs is to be established and widely adopted, thresholds for terminating AS and implementing definitive intervention need to be clearly identified. Additional data from long-term prospective registries and clinical trials with standardized indications for delayed intervention are needed to establish AS as an effective management strategy for SRMs. Furthermore, because clinical and radiographic characteristics on patient presentation are poor predictors of future growth rate, alternative measures for potential disease progression are needed (29, 30). With this in mind, the routine use of percutaneous renal mass biopsy has been suggested to improve risk stratification prior to initiating AS. However, this approach has not been shown to improve patient treatment selection or decrease the need for delayed intervention in AS series.

## Conclusion

AS may be an appropriate option for carefully selected patients with SRMs. Current data, however, suggest that delayed treatment is pursued in a significant percentage of patients within 3 years. Our pooled analysis reveals that in addition to tumor growth rate, a significant proportion of patients undergo delayed surgical therapy due to patient preference or anxiety in the absence of clinical progression. Additional data from long-term prospective registries and clinical trials with standardized indications for delayed intervention are needed to create a comprehensive AS protocol for SRMs.

## Conflicts of interest

The authors declare no potential conflicts of interest with respect to research, authorship and/or publication of this article.
